# Living with high output

**DOI:** 10.1007/s12471-012-0269-7

**Published:** 2012-03-20

**Authors:** L. C. Otterspoor, P. R. Stella

**Affiliations:** Department of Intensive care, University Medical Center Utrecht, Heidelberglaan 100, PO Box 85500, 3508 GA Utrecht, the Netherlands

**Fig. 1:**
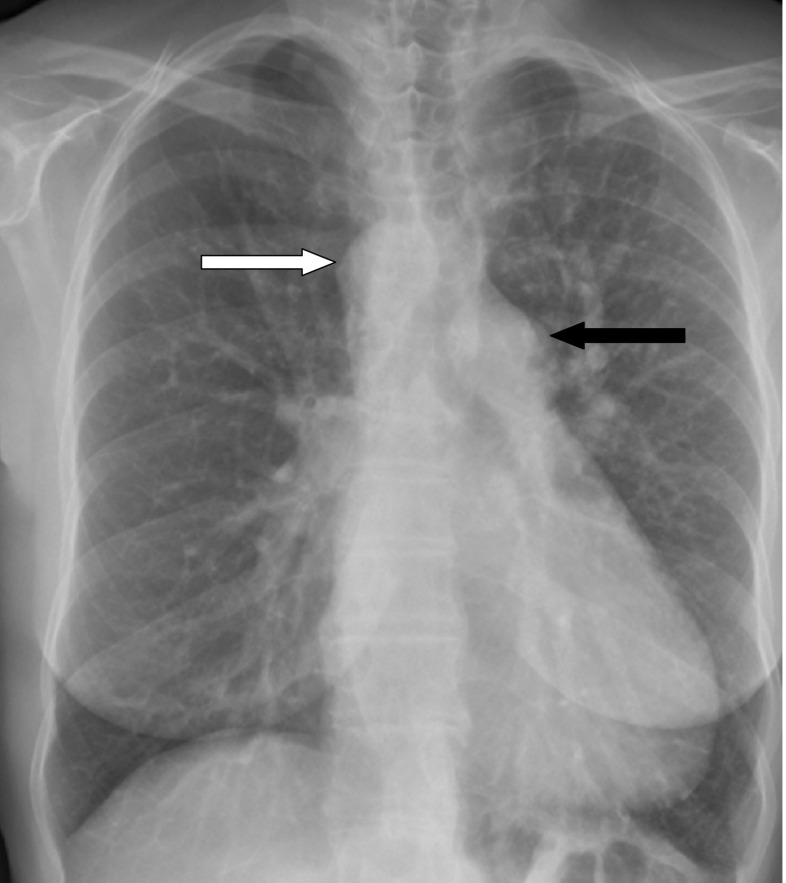
Chest radiography showing an enlarged heart figure, a right descending aorta (white arrow) and an enlarged left pulmonary artery (*black arrow*)

**Fig. 2:**
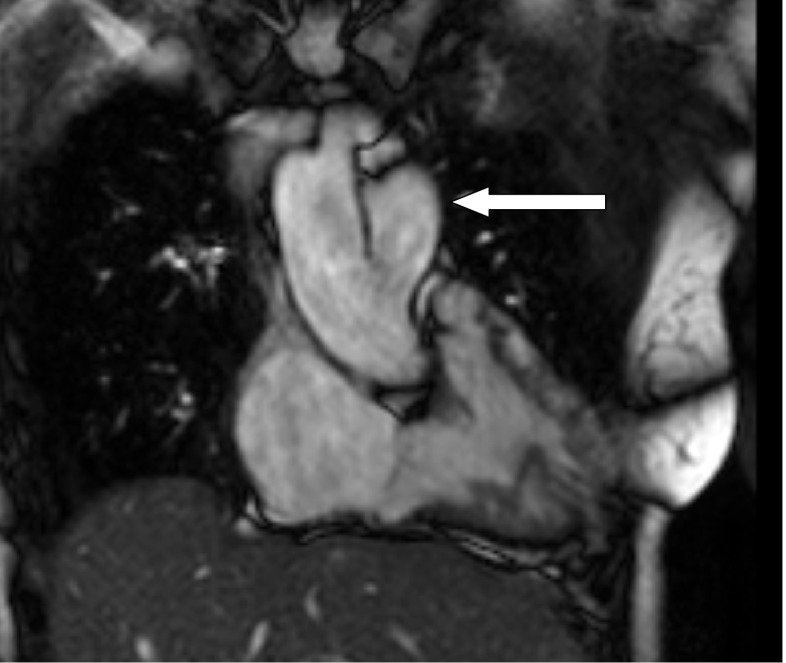
Magnetic resonance imaging showing the left pulmonary artery originating from the ascending aorta (*arrow*)

A 59-year-old woman presented for evaluation of heart failure. As a child she was known to have a ventricular septum defect which was no longer demonstrated during adulthood. She was normally in good shape but experienced yearly episodes of left-sided lower airway infections. During the last episode her general physician heard an ejection murmur and rales over her lungs. Chest radiography revealed an enlarged heart, a right descending aorta and a prominent left pulmonary artery (Fig. 1). There were no signs of congestion. Echocardiography revealed a slightly dilated left ventricle with an ejection fraction of 43%. Magnetic resonance imaging showed an anomalous left pulmonary artery originating from the right descending aorta (Fig. 2). The heart also appeared to be enlarged and the ejection fraction was slightly higher at 52%. Moreover, a high cardiac output of 11.6 l per minute was measured.

A pulmonary artery originating from the ascending aorta is a rare entity. The condition is described in children in whom surgical repair is attempted at an early age in order to avoid pulmonary hypertension [1, 2]. Our patient obviously had left-sided pulmonary hypertension from which she did not experience any symptoms, although it may have caused her repeated respiratory tract infections.

Because of a decreased afterload in combination with an increased pulmonary venous return, a doubling of the cardiac output ensued, likely leading to high output heart failure. This 59-year-old patient truly lived with a striking level of cardiac output, which had probably been present during her whole life, even though she had a normal exercise tolerance and played tennis regularly. Therefore, at this point no further treatment was installed. If progressive worsening of the left ventricular function develops, then pneumonectomy or banding of the pulmonary artery can be considered.

In a case of unexplained high output heart failure, looking for shunting, even at unexpected places, may be valuable.
